# Comprehensive screening for drugs that modify radiation-induced immune responses

**DOI:** 10.1038/s41416-021-01688-0

**Published:** 2022-02-19

**Authors:** Masayuki Okumura, Junyan Du, Shun-Ichiro Kageyama, Riu Yamashita, Yumi Hakozaki, Atsushi Motegi, Hidehiro Hojo, Masaki Nakamura, Yasuhiro Hirano, Yusuke Okuma, Hitomi S. Okuma, Katsuya Tsuchihara, Tetsuo Akimoto

**Affiliations:** 1 Department of Radiation Oncology, National Cancer Center Hospital East, Chiba, Japan; 2 Department of Radiology, Nagoya University Graduate School of Medicine, Nagoya, Aichi Japan; 3 Division of Translational Informatics, Exploratory Oncology Research and Clinical Trial Center, National Cancer Center, Chiba, Japan; 4 Department of Integrated Biosciences, Graduate School of Frontier Sciences, The University of Tokyo, Chiba, Japan; 5 Division of Radiation Oncology and Particle Therapy, Exploratory Oncology Research and Clinical Trial Center, National Cancer Center, Chiba, Japan; 6 Department of Thoracic Oncology, National Cancer Center Hospital, Tokyo, Japan; 7 Department of Breast and Medical Oncology, Clinical Research Support Office, National Cancer Center Hospital, Tokyo, Japan

**Keywords:** Tumour immunology, Drug screening

## Abstract

**Background:**

Combination therapy based on radiotherapy and immune checkpoint inhibitors (ICIs) was recently reported as effective for various cancers. The radiation-induced immune response (RIIR) is an essential feature in ICI-combined radiotherapy; however, the effects of drugs used concomitantly with RIIR remain unclear. We screened for drugs that can modify RIIR to understand the mutual relationship between radiotherapy and combined drugs in ICI-combined radiotherapy.

**Methods:**

We established a high-throughput system with reporter gene assays for evaluating RIIR, focusing on factors acting downstream of the STING-IRF pathway, which can stimulate cancer cells, T cells, and dendritic cells. We further quantified the effects of 2595 drugs, including those approved by the Food and Drug Administration, on RIIR in vitro.

**Results:**

The reporter assay results correlated well with the expression of immune response proteins such as programmed death-ligand 1. This high-throughput system enabled the identification of drugs including cytotoxic agents, molecular-targeted agents, and other agents that activate or suppress RIIR.

**Conclusions:**

Our study provides an encyclopedic catalogue of clinically approved drugs based on their effect on RIIR. In ICIs combined radiotherapy, activation of STING-IFN may improve the therapeutic effect and our result could form a biological basis for further clinical trials combining radiotherapy with ICIs.

## Graphical abstract

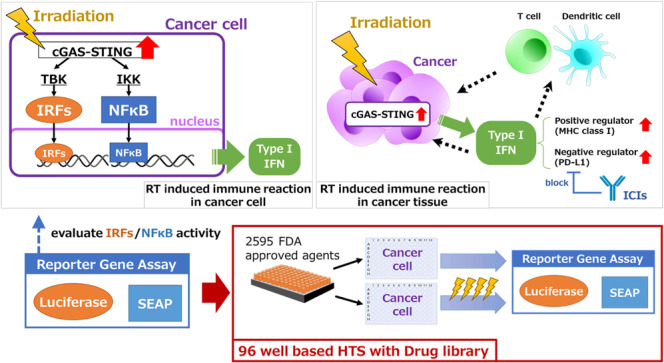

## Background

Radiotherapy (RT) is a widely used treatment for various cancers, including lung cancer [1], and recent data revealed that RT can enhance the efficacy of immune checkpoint inhibitors (ICIs) by upregulating MHC class I and programmed death-ligand 1 (PD-L1) expression in cancer cells [2]. Furthermore, a phase III trial showed that definitive chemoradiotherapy (CRT) followed by administration of durvalumab, a PD-L1 antibody, prolonged overall survival in patients with locally advanced non-small-cell lung cancer (NSCLC) [3, 4].

Several drugs have also been reported to modify the cancer immune response; for example, corticosteroids suppress the effects of ICI monotherapy [5, 6]. It was recently reported that in combination therapy using RT, the effect of ICI combined with RT differed depending on the cytotoxic drug used concomitantly [7]. In CRT for NSCLC, it is possible to select and use two anticancer drugs in regimens known as platinum doublets, in which a platinum agent such as cisplatin is paired with a third-generation chemotherapy counterpart such as a taxane, pemetrexed, etoposide (ETP), vinorelbine (VNR), or 5-fluorouracil (5-FU) [8–10]. Therefore, it is important to evaluate whether cytotoxic drugs combined with RT enhance the immune response. For instance, molecular-targeted drugs such as olaparib and trametinib are likely to induce immune responses against cancer [11].

In addition to anticancer drugs, many patients with cancer are prescribed several other medicines such as COX inhibitors for anti-inflammatory purposes during supportive care. Therefore, it is also essential to investigate the effects of drugs other than anticancer drugs on the immune response. However, as over 2000 drugs are currently approved, it is difficult to screen the effect of all drugs on the radiation-induced immune response (RIIR) through in vivo experiments.

Recently, radiation was shown to induce an immune reaction in the tumour tissue and surrounding immune cells via the micronucleus-forming STING-type I interferon (IFN) pathway in vitro and in vivo, which is considered as a master regulator of the cancer-immune reaction including the immune reaction in the tumour microenvironment (TME) triggered by irradiation [12]. STING activation enhances cancer antigen presentation, contributes to the priming and activation of T cells, facilitates the trafficking and infiltration of T cells into tumours, and promotes the recognition and killing of cancer cells by T cells [13, 14]. Notably, radiation activates the STING-IRFs-IFN pathway in cancer cells and then activates surrounding immune cells [12]. Therefore, evaluation of radiation-induced STING-IRFs-IFN pathway activation in cancer cells in vitro can provide a potential indicator of the RIIR. Hence, we established a high-throughput system based on a reporter gene assay (RGA) for easy evaluation of the RIIR and screened the effect of a library of 2595 clinically approved agents on the RIIR. We also assessed the potential underlying mechanisms of their effects on the RIIR.

## Methods

### Cell lines and RGA

The A549 cell line is an NSCLC cell line that is often used in radiation experiments [15]. Its radiation resistance is moderate, and even a clinical dose of 2 Gy/fr shows low toxicity; therefore, we used this cell line in our experiments. The A549-Dual cell line was obtained from Invitrogen (Carlsbad, CA, USA). The cell culture, luciferase assay, and secreted alkaline phosphatase (SEAP) RGA were performed according to the A549-Dual instructions (https://www.invivogen.com/a549-dual) [16].

STING-knockout A549 cells were established using The TrueCut Cas9 protein v2 and *STING* gRNA(CRISPR802251_SG and CRISPR802254_SG) as performed previously (unpublished).

### In vitro irradiation

**Fig. 1:**
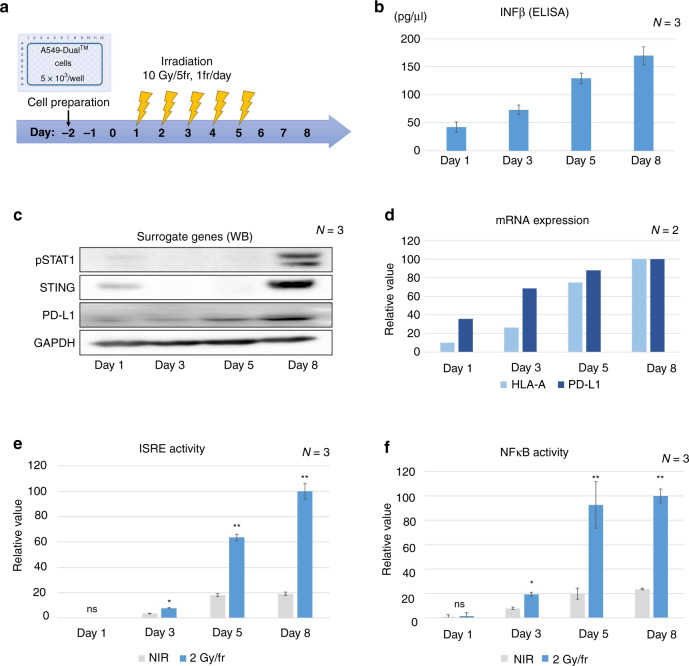
Assessment of radiation-induced immune response in A549-Dual cells. **a** Irradiation schedule. **b** Quantification of IFNβ secretion during and after irradiation using ELISA. **c** Protein expression of STAT1Y701, STING, and PD-L1 during and after irradiation. **d** mRNA expression of HLA-A and PD-L1 during and after irradiation. Expression levels at each time point were calculated relative to that on day 8 (percentage) (2 days post 10 Gy/5 fr irradiation). **e**, **f** Reporter gene assay assessing ISRE and NF-κB activities in irradiation and non-irradiation groups. Activities at each time point were calculated relative to that on day 8 (percentage). Error bars represent STDEV. *P < 0.01, **P < 0.001, ns, not significant, as determined by unpaired Student’s *t*-test between NIR with IR group. WB western blotting, IFN interferon, ISRE interferon-stimulated response element, NIR non-irradiated.

A549-Dual cells were cultured in 96-well plates prior to irradiation as previously described [17]. The cells were irradiated at a dose rate of 3 Gy/min using a Clinac iX System Linear Accelerator (Varian Medical Systems, Palo Alto, CA, USA) with an energy of 6 MV photons. The irradiation plan was performed by simulated computed tomography and pinnacle. The dose distribution was prepared in the range of D90 and irradiated at a dose rate of 600 cGy/min. The cells were exposed to a total dose of 10 Gy in five times per week. Figure 1a shows a schematic of the cell culture and irradiation regimen.

### Enzyme-linked immunosorbent assay (ELISA)

We measured the concentration of IFN-β on day 1 (before irradiation), day 3 (24 h after 4 Gy/2 fractions [fr]), day 5 (24 h after 8 Gy/4 fr), and day 8 (72 h after 10 Gy/5 fr).　The IFN-β concentration was measured in 100 μL of culture medium using Human IFN-β ELISA Kit (R&D Systems, Minneapolis, MN, USA) following the manufacturer’s protocol.

### Drug screening

Detailed information regarding the library of chemical drugs (Selleck Chemicals, Houston, TX, USA, L2000-Z383425) and screening results are listed in Supplementary Table 1. This chemical library classified drugs into categories such as cancer, inflammation, and infection and identified target molecules. Screening was performed in a 96-well plate using a semi-automatic INTEGRA VIAFLO 96 system (INTEGRA Biosciences, Tokyo, Japan). The test drug was added at a final concentration of 10 µM at 1 h before 8 Gy/4 fr irradiation. Anticancer drugs, anti-inflammatory drugs, and other drugs selected in the initial screening were revalidated at concentrations of 10–0.1 µM [18], and the reproducibility of the results was confirmed in at least two independent experiments. Drugs that were more toxic than the 30% inhibition concentration (IC_30_) at 0.1 μM were screened again at up to 1 nM. The results for the non-irradiated and irradiated groups are shown as the fold-change with respect to non-irradiated and irradiated cells treated with vehicle only, respectively. Cytotoxicity was assessed and corrected using the alamar blue assay. Concentrations at which the survival rate was 10% or less were excluded (Supplementary Table 1).

### Gene expression analysis

RNA was extracted from 10^6^ to 10^7^ irradiated cancer cells using an RNeasy mini kit (QIAGEN, Hilden, Germany). The quality of the extracted RNA was assessed on an Agilent Bioanalyzer 2100 system (Agilent, Santa Clara, CA, USA), and RNA sequencing (RNA-seq) was performed (Annoroad, Beijing, China). All data were subjected to quality control filtering, trimming, and adaptor removal using the FASTQC and FASTQ toolkits (BaseSpace, Illumina, San Diego, CA, USA). Specifically, reads containing *N* > 10% (where *N* indicates an undetermined base) or of low quality (*Q* score ≤ 5), which was over 50% of the total bases, were removed. All filtered sequences were aligned to the hg38 reference genome used for gene expression analyses and represented as transcripts per million calculated using RSEM software [19]. Differentially expressed genes (DEGs) were identified using the R package edgeR [20] with a false discovery rate ≤ 0.05. DEGs were used for Gene Ontology (GO) and Kyoto Encyclopedia of Genes and Genomes (KEGG) analyses using the iPathway Guide (ADVAITA, Ann Arbor, MI, USA) (Supplementary Tables 2 and 3) and Metascape [21] and then visualised utilising Cytoscape [22].

Principal component analysis (PCA) was performed using R package plot3D, with the transcripts per million of all expressed genes in each sample used as input. A Venn diagram was constructed using the web tool InteractiVenn [23].

### Nuclear staining

Cells were fixed in 4% formaldehyde for 15 min at 24 °C, washed with phosphate-buffered saline, and stained with 1 µg/mL Hoechst 33342 for 10 min at room temperature. Images were captured using a BZ9000 fluorescence microscope system (Keyence, Tokyo, Japan). The structures stained by Hoechst 33342 outside the nucleus were classified as micronuclei. Cells with micronuclei were counted manually in each field (×20 magnification), and the results are expressed as the percentage of all cells within the field counted. A total of 50–70 cells was counted in each field.

### Real-time polymerase chain reaction (PCR)

The mRNA expression levels of *PDL1* and *MX1* were quantified by performing real-time PCR and using specifically designed primers. The following primers were used: *PDL1* forward 5′-GGTGGTGCCGACTACAAGCGA-3′, *PDL1* reverse 5′-CCTTGGGTAGCCCTCAGCCT-3′; *MX1* forward 5′-TCTGAGGAGAGCCAGACGAT-3′, *MX1* reverse 5′-ACTCTGGTCCCCAATGACAG-3′; *HLA-B* forward 5′-GCGAGTCCCGAGGATGGC-3′, *HLA-B* reverse 5′-TTGTAGTAGCCGCGCAGGT-3′; *ACTB* forward 5′-TCACCCACACTGTGCCCATCTACGA-3′, *ACTB* reverse 5′-CAGCGGAACCGCTCATTGCCAATGG-3′. The mRNA expression levels were quantified based on the relative cycle threshold values for each sample and normalised to beta-actin (*ACTB*) expression.

### Western blotting (WB)

Cells were harvested 24 h after exposure to the radiation doses indicated. For protein extraction, cells were scraped and washed with cold PBS, then lysed in RIPA buffer (Wako, Japan) containing a proteinase inhibitor cocktail (Sigma, P8340) for 20 min on ice and cleared by centrifugation. The protein samples were quantified by a BCA assay (Thermos), separated by SDS-PAGE (Wako, Japan), transferred to a PVDF membrane (Bio-Rad), and detected by immunoblotting with the antibodies indicated (Supplementary Table 4). More specifically, the blots were detected with ECL reagent (GE Healthcare, RPN2232) and visualised by ImageQuant LAS 4000mini (GE). Relative quantification was performed using ImageJ software, and GAPDH was used as the reference for normalisation.

## Results

### RGA-based validation of RIIR assessment in A549-dual cells

We investigated the validity of assessing the RIIR in cancer cells by performing an RGA. A549-Dual cells have been widely reported as reporter cells for IFN-stimulated response element (ISRE) and NF-κB activity [24]. First, we irradiated the NSCLC cell line A549-Dual at a dose of 10 Gy in 5 fr as a preclinical model and evaluated the RIIR (Fig. 1a). The protein expression of STATY701 and IFN-β, which have been reported as surrogates for the RIIR [25, 26], was assessed using ELISA and western blotting (Fig. 1b, c). Furthermore, we investigated the mRNA expression of the MHC class and PD-L1 genes that are downstream of STING; these genes directly participate in the cancer-immune response and are biomarkers of immune checkpoint inhibition [7, 27]. The expression of surrogate genes and MHC class I (HLA-A and HLA-B) and PD-L1 mRNAs was correlated with the activities of ISRE and NF-κB (Fig. 1b–f). All immune responses increased gradually at day 3 (i.e., after 4-Gy irradiation) and were maintained or increased up to day 8. GO analysis also showed that type I IFN and NF-κB signalling pathways were upregulated significantly from days 3 and 5, respectively (Supplementary Table 2). Thus, we inferred that the results of RGA accurately reflected the RIIR in cancer cells. Finally, we confirmed in STING-knockout experiments that ISRE and NF-κB activity reflected STING activity induced by IR (Supplementary Fig. 1). Knocked out of STING significantly reduced radiation-induced ISRE and NFKB activity (*P* = 3e^−5^ and *P* = 5e^−6^, respectively, student *t-*test).

### Drug screening using a library of Food and Drug Administration (FDA)-approved drugs

**Fig. 2:**
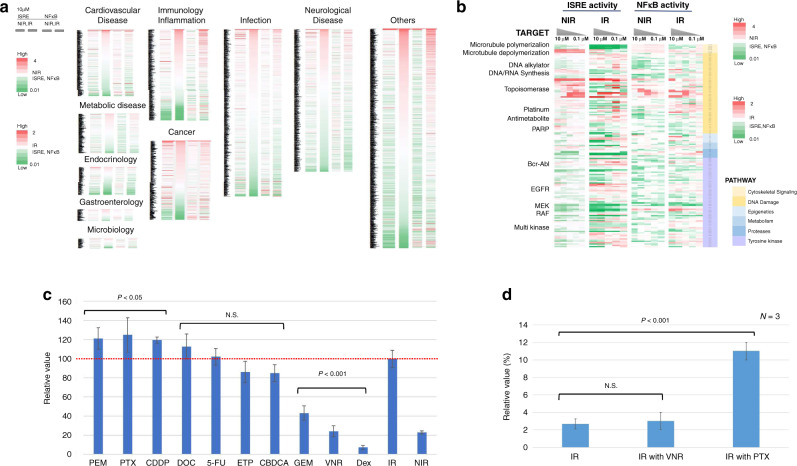
Modification of the immune response by FDA-approved agents. **a** Heat map showing ISRE and NF-κB activities after administering 2595 FDA-approved agents at concentrations of 10 μM. Fold-change is shown with respect to the untreated group*.* **b** ISRE and NF-κB activities after administering 233 agents associated with cancer at a concentration range of 10–0.1 μM. Fold-change is shown with respect to the untreated group. **c** ISRE activities after administering the drugs to non-small cell lung cancer cells irradiated at 8 Gy/4 fr. Values are expressed relative to the activity of the IR-alone (drug-free) group as 100. **d** Percentage of micronuclei formation after administering vinorelbine or paclitaxel with or without 8 Gy/4 fr irradiation. Error bars represent STDEV. ns, not significant, as determined by unpaired Student’s *t*-test between IR-alone with IR with the drug group. ISRE interferon-stimulated response element, NIR non-irradiated, IR irradiated, PEM pemetrexed, PTX paclitaxel, CDDP cisplatin, DOC docetaxel, CBDCA carboplatin, ETP etoposide, GEM gemcitabine, VNR vinorelbine, Dex dexamethasone.

To investigate the effects of chemical drugs on the RIIR, we individually treated A549-Dual cells with 2595 FDA-approved drugs (10 µM each), followed by irradiation at a dose of 8 Gy/4 fr. As there are technical difficulties in creating an irradiated and a non-irradiated group on the same plate, the results for the non-irradiated and irradiated group are shown as the fold-change with respect to non-irradiated and irradiated cells treated with vehicle only, respectively. Cytotoxicity was assessed and corrected using the alamar blue assay. Concentrations at which the survival rate was 10% or less were excluded. Figure 2a shows the ISRE and NF-κB activities of the cells treated with 2595 drugs in the irradiated and non-irradiated groups. Most anticancer drugs exert effects at blood concentrations of 1–10 μM, whereas some function at concentrations around 0.1 μM [18]. For detailed profiling, the immune response was evaluated in cancer cells treated with 233 drugs at concentrations of 0.1–10 μM (Fig. 2b). Experiments were performed at least twice to verify the reproducibility of the results. Immune response modulation by given drugs differed according to the chemotherapeutic drugs used; in other words, some drugs activated, whereas others suppressed the RIIR (Fig. 2c). Specifically, the immune response was significantly increased by pemetrexed (PEM), paclitaxel (PTX) and cisplatin (CDDP) compared to irradiation alone. The immune response was maintained by docetaxel (DOC), carboplatin (CBDCA), 5-FU, and ETP. In contrast, the immune response was significantly inhibited compared to irradiation alone (by more than 50%) in response to treatment with the pyrimidine metabolism antagonist gemcitabine (GEM), microtubule polymerisation inhibitor such as VNR, and a corticosteroid such as dexamethasone (Dex) (Fig. 2c). The validity of the RGA results was evaluated by comparing the changes in mRNA expression levels of *MX1*, which is regulated by the ISRE promoter, and the expression of *PDL1* and MHC class I (HLA-A and HLA-B), which are regulated by STING. The RGA results correlated well with the mRNA expression levels of *MX1* and *PDL1* observed in three independent real-time polymerase chain reaction experiments (Supplementary Fig 2A). The RGA results also correlated with the levels of MHC class I mRNA expression in two independent RNA-seq (Supplementary Fig 2B).

### Transcriptome analysis by RNA-seq

Next, we investigated the mechanisms underlying the differential modifications of the RIIR according to the drugs used. As described above, the microtubule depolymerisation inhibitors PTX and DOC resulted in stronger activation of the RIIR than that observed with the microtubule polymerisation inhibitor VNR when combined with irradiation. Micronuclei formation, which reflects activation of the STING-type I IFN pathway, was more prominent following PTX treatment than after VNR treatment when combined with radiation (Fig. 2d and Supplementary Fig 3A). Furthermore, we found that CDK1 and Aurora A inhibitors, which arrest the cell cycle before metaphase, attenuated the RIIR. In contrast, Aurora B inhibitors, which arrest the cycle from metaphase to telophase, did not significantly influence the RIIR (Supplementary Fig 3B).

**Fig. 3:**
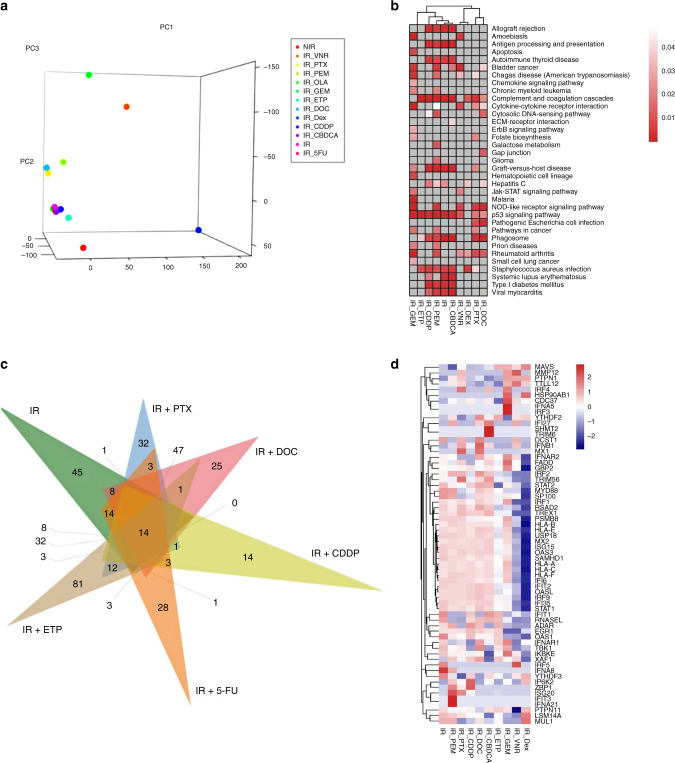
Transcriptome analysis of the radiation-induced gene expression profile. **a** PCA plot analysis in NIR, IR, IR with PEM, PTX, CDDP, DOC, CBDCA, ETP, GEM, VNR, 5-FU, OLA, and Dex groups. **b** KEGG enrichment analysis for signal pathway in IR, IR with PEM, PTX, CDDP, DOC, CBDCA, ETP, GEM, VNR, and Dex groups. **c** Venn diagram of biological process in IR, IR with DOC, PTX, CDDP, 5-FU, and ETP groups. **d** Heat map of gene expression profiles in IR, IR with PEM, PTX, CDDP, DOC, CBDCA, ETP, GEM, VNR, and Dex groups. NIR non-irradiated, IR irradiated, IR with PEM pemetrexed, PTX paclitaxel, CDDP cisplatin, DOC docetaxel, CBDCA, carboplatin, ETP etoposide, GEM gemcitabine, VNR vinorelbine, 5-FU 5-fluorouracil, OLA olaparib, Dex dexamethasone.

Next, we analysed the effect of chemotherapeutic drugs that are typically used with RT as platinum doublets and Dex in CRT for NSCLC on the gene expression profile by performing RNA-seq. The gene expression profiles were analysed and compared in the following groups: non-irradiation, irradiation alone, and irradiation combined with each drug. For each drug, 3 μM (PEM, ETP, 5-FU, OLA), 2 μM (PTX, DOC), 1 μM (GEM, CDDP, CBDCA), and 0.1 μM (VNR, DEX) were adopted because these concentrations were the IC_50_ dose in A549 cells and within the range of the blood concentration in patients. The total gene expression profile is shown PCA plot (Fig. 3a), and as the KEGG pathway (Fig. 3b) and GO (Fig. 3c and Tables 1 and 2, and Supplementary Table 3) analysis results. In KEGG enrichment analysis for signal pathways, the p53 signalling pathway (hsa04115) was significantly enriched in combination with most drugs (Fig. 3b). In cluster analysis on the KEGG pathway, irradiation alone and the combination with PEM, CDDP, and CBDCA formed the same cluster. PTX and DOC also formed other clusters. Table 2 and Supplementary Table 3 showed the outline of the biological process induced by irradiation in combination with each drug and 14 common biological processes (Fig. 3c), including type I IFN signalling except for GEM, VNR, and Dex (Table 1 and Supplementary Table 3), were confirmed. We also show the expression profile of genes involved in type I IFN signalling in a heat map (Fig. 3d).

**Table 1:** Enrichment analysis of genes upregulated in response to drug-combined radiation in the non-irradiated group.

Drug	*P-*value	*Q*-value	Gene ID (type I interferon signalling pathway, GO: 0060337)
IR	1.85E−26	2.06E−23	IFI6/ISG15/HLA-B/HLA-A/HLA-C/IFIT1/IFIT3/OASL/IFITM1/IFI27/IFIT2/OAS3/MX1/STAT1/IFI35/HLA-F/HLA-E/OAS1/ADAR/XAF1/STAT2/PSMB8
PEM	4.78E−14	4.82E−11	HLA-B/ADAR/IFI27/IFIT3/OASL/IFIT2/HLA-C/ISG15/IFIT1/HLA-F/IFI6/HLA-A/STAT1
PTX	5.59E−06	0.000695	HLA-B/IFIT3/IFIT2/OASL/HLA-F/ISG15
CDDP	2.46E−22	2.24E−19	ADAR/HLA-B/IFIT3/OASL/ISG15/IP6K2/IFIT2/HLA-C/IFI6/IFI27/XAF1/STAT1/IFIT1/HLA-A/MX1/HLA-F/OAS3
DOC	1.13E−11	6.69E−09	IFIT3/HLA-B/OASL/IFIT2/ADAR/IP6K2/IFIT1/ISG15/IFI27/STAT1
CBCDA	1.31E−23	1.46E−20	ISG15/IFI6/HLA-B/HLA-C/HLA-A/IFIT3/OASL/IFI27/IFIT1/IFIT2/IFITM1/MX1/OAS3/STAT1/HLA-F/HLA-E/OAS1/IFI35/XAF1/TREX1
ETP	0.000337	0.005645	IFI6/HLA-A/ADAR/HLA-B/HLA-C
GEM	n.s.		
VNR	n.s.		
Dex	n.s.		

The dynamics of type I interferon signal analysis are shown.

*IR* irradiated, *PEM* pemetrexed, *PTX* paclitaxel, *CDDP* cisplatin, *DOC* docetaxel, *CBDCA* carboplatin, *ETP* etoposide, *GEM* gemcitabine, *VNR* vinorelbine, *Dex* dexamethasone, *n.s.* not significant.

**Table 2:** Gene ontology analysis in IR, IR with PEM, PTX, CDDP, DOC, CBDCA, and ETP groups.

**IR**								
**Description**	**Gene ratio**	***q*-value**						
Type I interferon signalling pathway	22/141	2.06E−23						
Cellular response to type I interferon	22/141	2.06E−23						
Response to type I interferon	22/141	4.012E−23						
Response to virus	26/141	1.383E−15						
Interferon-gamma-mediated signalling pathway	13/141	2.653E−10						
Defence response to virus	18/141	4.548E−10						
Response to interferon-gamma	16/141	4.747E−10						
Cellular response to interferon-gamma	15/141	8.889E−10						
Defence response to other organisms	23/141	3.836E−09						
Regulation of viral life cycle	12/141	5.747E−07						
**IR_PEM**			**IR_PTX**			**IR_CDDP**		
**Description**	**Gene ratio**	***q*-value**	**Description**	**Gene ratio**	***q*-value**	**Description**	**Gene ratio**	***q*-value**
Type I interferon signalling pathway	13/115	4.818E−11	Response to virus	13/82	1.031E−05	Type I interferon signalling pathway	17/87	2.2376E−19
Cellular response to type I interferon	13/115	4.818E−11	Regulation of viral genome replication	8/82	1.658E−05	Cellular response to type I interferon	17/87	2.2376E−19
Response to type I interferon	13/115	5.793E−11	Viral genome replication	8/82	7.319E−05	Response to type I interferon	17/87	3.3399E−19
Response to virus	19/115	4.034E−10	Negative regulation of viral genome replication	6/82	0.000155	Response to virus	20/87	1.0874E−13
Regulation of viral genome replication	9/115	6.746E−06	Regulation of viral life cycle	8/82	0.000185	Regulation of viral genome replication	10/87	2.0185E−08
Intrinsic apoptotic signalling pathway by p53 class mediator	8/115	1.644E−05	Cellular response to lipopolysaccharide	8/82	0.0005666	Regulation of viral life cycle	11/87	6.2425E−08
Regulation of viral life cycle	10/115	1.679E−05	Regulation of inflammatory response	11/82	0.0005666	Defence response to virus	13/87	6.2425E−08
Response to mineralocorticoid	6/115	1.732E−05	Cellular response to molecule of bacterial origin	8/82	0.0005794	Cellular response to interferon-gamma	11/87	9.6465E−08
Mitotic cell cycle checkpoint	10/115	1.817E−05	Negative regulation of viral life cycle	6/82	0.0005794	Negative regulation of viral genome replication	8/87	1.0189E−07
Regulation of viral process	11/115	2.351E−05	Viral life cycle	10/82	0.0006948	Response to interferon-gamma	11/87	2.1916E−07
			Type I interferon signalling pathway	6/82	0.0006948			
**IR_DOC**			**IR_CBDCA**			**IR_ETP**		
**Description**	**Gene ratio**	***q*-value**	**Description**	**Gene ratio**	***q*-value**	**Description**	**Gene ratio**	***q*-value**
Response to virus	16/77	2.362E−09	Type I interferon signalling pathway	20/135	1.463E−20	Intrinsic apoptotic signalling pathway	15/110	8.8923E−07
Type I interferon signalling pathway	10/77	6.689E−09	Cellular response to type I interferon	20/135	1.463E−20	Intrinsic apoptotic signalling pathway by p53 class mediator	9/110	1.7839E−06
Cellular response to type I interferon	10/77	6.689E−09	Response to type I interferon	20/135	2.547E−20	Mitotic DNA damage checkpoint	9/110	3.4334E−06
Response to type I interferon	10/77	7.852E−09	Response to virus	22/135	8.958E−12	Mitotic DNA integrity checkpoint	9/110	3.4334E−06
Regulation of viral genome replication	9/77	1.617E−07	Response to interferon-gamma	15/135	4.663E−09	Mitotic G1 DNA damage checkpoint	8/110	3.4334E−06
Viral genome replication	9/77	1.166E−06	Cellular response to interferon-gamma	14/135	9.2E−09	Mitotic G1/S transition checkpoint	8/110	3.4334E−06
Regulation of viral life cycle	9/77	5.169E−06	Interferon-gamma-mediated signalling pathway	11/135	4.93E−08	G1 DNA damage checkpoint	8/110	3.4334E−06
Regulation of viral process	10/77	6.635E−06	Negative regulation of viral genome replication	9/135	2.127E−07	Regulation of peptidase activity	15/110	9.4432E−06
Regulation of symbiosis, encompassing mutualism through parasitism	10/77	2.051E−05	Regulation of viral genome replication	10/135	1.088E−06	Mitotic cell cycle checkpoint	10/110	1.3216E−05
Negative regulation of viral genome replication	6/77	3.328E−05	Regulation of viral life cycle	11/135	4.208E−06	Intrinsic apoptotic signalling pathway in response to DNA damage by p53 class mediator	6/110	6.9816E−05
						Type I interferon signalling pathway	5/110	0.00564458

The results of the top 10 significantly enriched categories and type I Interferon signalling pathways are shown.

*IR* irradiated, *PEM* pemetrexed, *PTX* paclitaxel, *CDDP* cisplatin, *DOC* docetaxel, *CBDCA* carboplatin, *ETP* etoposide.

The molecular-targeted drugs analysed enhanced or suppressed the RIIR, similar to the effects of chemotherapeutic drugs. For example, osimertinib which specifically inhibits epidermal growth factor receptor (EGFR), entrectinib which inhibits proto-oncogene tyrosine-protein kinase/tropomyosin receptor kinase (ROS1/NTRK), crizotinib which inhibits MET/anaplastic lymphoma kinase (MET/ALK), and olaparib which inhibits poly ADP ribose polymerase (PARP) enhanced or maintained the RIIR, whereas dabrafenib which inhibits proto-oncogene B-Raf (BRAF), trametinib which inhibits mitogen-activated protein kinase (MEK), and vorinostat which inhibits histone deacetylase (HDAC) significantly reduced the RIIR (Supplementary Fig. 4). Anti-inflammatory and immunological drugs such as corticosteroids, JAK inhibitors, antimetabolites, and microtubule polymerisation inhibitors suppressed the RIIR (Supplementary Fig. 5). In contrast, COX inhibitors, which are often used in patients with cancer, had little effect on the RIIR (Supplementary Fig. 5).

## Discussion

We used a high-throughput RGA system to quantify the effects of clinically used drugs on the RIIR. Many drugs that activate, maintain, or suppress the RIIR were screened (Supplementary Table 1). Below, we discuss the biological basis and clinical importance of the findings of the current study.

The RIIR can be either STING-dependent or independent, but most responses require gene expression via the IRF-dependent ISRE promoter or NF-κB promoter [12, 26]. Our high-throughput RGA system was validated by the results showing that ISRE and NF-κB activity correlated with IFNβ secretion and mRNA expression levels of immune-related factors, including *MX1* regulated by the ISRE promoter and *PDL1*, HLA-A, and HLA-B regulated by STING (Fig. 1 and Supplementary Fig. 2 and Supplementary Table 2). Furthermore, using the STING-knockout cell line, we confirmed that approximately 95% of radiation-induced ISRE activity and 70% of NF-κB activity was derived from STING (Supplementary Fig. 1).

The drug screening results revealed that chemotherapeutic drugs used in combination with RT maintained or suppressed the RIIR, depending on their targets. Microtubule-targeting drugs, including PTX, DOC, and VNR, had particularly distinct effects, despite being M-phase inhibitors (Fig. 2b, c). These drugs can be selected as partners of platinum doublets and are used in combination with ICIs. The large difference in activation or suppression of the STING-IRFs-IFN pathway according to each drug is an interesting finding, as the efficacy of the combination of ICIs and RT may be influenced via modification of the RIIR.

Chemotherapeutic drugs induce micronuclei formation, although the degree of this induction differs according to the targets [28, 29]. In addition, antimitotic drugs can cause micronuclei formation, induce a cancer immune reaction, and activate lymphocytes by activating the STING-IRFs-IFN in cancer cells both in vitro and in vivo [30]. Our study identified the RIIR-modulating effects of these antimitotic drugs. For example, M-phase inhibitors (agents that target processes before the metaphase), such as CDK1 inhibitor, Aurora A, and VNR, strongly suppressed the RIIR. In contrast, drugs that target processes after metaphase, such as Aurora B, DOC, and PTX, maintained or enhanced the RIIR (Supplementary Fig. 3B). The formation of micronuclei differed significantly in response to VNR and PTX treatment, which may differentially modulate the RIIR.

The large difference in the RIIR, mediated by different cytotoxic drugs commonly used in CRT for NSCLC, was the most interesting finding of the current study. Platinum-based chemotherapy, including platinum doublet, has been established as a standard chemotherapy regimen of CRT for NSCLC [8–10]; however, its routine use remains controversial in combination with ICIs [31] because of the effect on the TME. Post-hoc analysis of the PACIFIC trial suggested that the clinical outcomes were slightly better in patients who received CRT with cisplatin-based chemotherapy than in those who received CRT with carboplatin-based chemotherapy [7]. Our results based on the high-throughput RGA system also revealed that the effect of cisplatin on the RIIR was higher than that of carboplatin, suggesting that chemotherapeutic drugs differentially affect the RIIR.

In gene expression analysis, PCA plots, KEGG pathway analysis, and GO analysis identified gene expression profiles and biological processes in irradiated cells treated with each drug (Fig. 3a, c). The immune reaction was commonly regulated via the IFN-dependent pathway (Fig. 3b and Table 1 and Supplementary Table 3). This finding indicates that the type I IFN pathway is a common and effective therapeutic target for platinum doublet combination RT.

Additionally, drugs specific for molecular targets, such as inhibitors of EGFR, PARP, BRAF, MEK, ALK/ROS, and HDAC, are part of a promising therapeutic approach for metastatic NSCLC and exhibited distinct effects on the RIIR (Supplementary Fig. 4). Furthermore, we identified several anti-inflammatory drugs that suppressed the anticancer immune response induced by irradiation (Supplementary Fig. 5). Although corticosteroids mainly affect the activity and function of lymphocytes and neutrophils and reduce the effects of ICI [5, 6], we observed that corticosteroids also modified the RIIR. Moreover, JAK inhibitors, MTX, and colchicine, which are used to treat autoinflammatory diseases, reduced the RIIR, although COX inhibitors had little effect. Importantly, COX inhibition and the IFN response have been reported to correlate with radiosensitivity [32]. However, recent studies reported that the radiation-induced IFN response is important for the abscopal effect, and the effect of radiation-induced IFN on cancer cells remains controversial regarding whether this effect is favourable for cancer treatment [33].

As the ISRE activity per surviving cell was measured using the alamar blue assay in our experiment, radiosensitivity requires further analysis. We discussed only the immune response per cell and the potential for radiation therapy with ICIs.

Herein, we focused only on the immune response of cancer cells to irradiation, which is a limitation of this study. It is well-known that immune cells, including lymphocytes, are influenced or inactivated by RT or CRT [34], indicating that the effect of RT on immune cells influences the overall immune response of cancer. However, it has been reported that radiation first activates the STING-IRFs-IFN pathway in cancer cells, and then in surrounding immune cells [12]. Therefore, evaluation of radiation-induced STING activity in cancer cells in vitro may reflect the RIIR in cancer cells. It has been reported that olaparib, a PARP inhibitor, activates tumour cells and surrounding lymphocytes [35], whereas steroids reduce the cancer immune response [5, 6]. Our system successfully reproduced the immune response enhancement mediated by olaparib and immune response suppression mediated by steroids, suggesting that this high-throughput RGA system can be used to evaluate the immune status while considering the TME. Furthermore, our screening system required stably established RGA cell lines, and A549 is the only NSCLC cell line that has been established. Therefore, we only used A549 cells in this study for initial screening; however, further studies are needed to investigate various NSCLC cell lines such as EGFR and EML-ALK mutant cell lines.

Although TME analysis of cancer tissue after irradiation or clinical data analysis of ICI-combined RT can provide more important information, pathological analysis and animal experiments are costly and time-consuming, and it is difficult to investigate the effects of more than 2000 approved drugs. Our high-throughput system is advantageous for providing primary results related to the effects of approved drugs on the RIIR in cancer cells. Our high-throughput system is complementary to TME analysis and can facilitate the advancement of ICI-combined RT. Further studies are needed to determine whether the differences in STING activation or suppression of cytotoxic and other drugs observed in this study affect the outcome of ICI combined with RT. CRT followed by PD-L1 antibody treatment for NSCLC began only two years ago, and data are currently insufficient for retrospective analysis but may be possible in the near future. Our findings would be useful for future retrospective analysis.

In summary, we established a high-throughput system for easily detecting the RIIR with STING activity and comprehensively investigated the effects of 2595 FDA-approved, clinically used drugs on RIIR in vitro using a high-throughput RGA system that can quantify the STING activity, which is an initial trigger of the RIIR. These results provide an encyclopedic catalogue of clinically approved drugs based on the effect on the RIIR. This catalogue can form the biological basis for further clinical trials of combining RT with ICIs.

## Supplementary information

Author’s consent English

Reproducibility checklist

Supplementary figures

Supplementary Table 1

Supplementary Table 2

Supplementary Table 3

Supplementary Table 4

## Data Availability

The datasets relevant to the current study are available from the corresponding author on reasonable request.
