# Supplementary material for: Comprehensive screening for drugs that modify radiation-induced immune responses
Source: Br J Cancer. 2022 Feb 19;126(12):1815–23. doi: 10.1038/s41416-021-01688-0 (PMC9174493; doi:10.1038/s41416-021-01688-0)
Supplement: Supplementary file 3 — Supplementary figures [file 41416_2021_1688_MOESM3_ESM.pptx]

## Slide 1
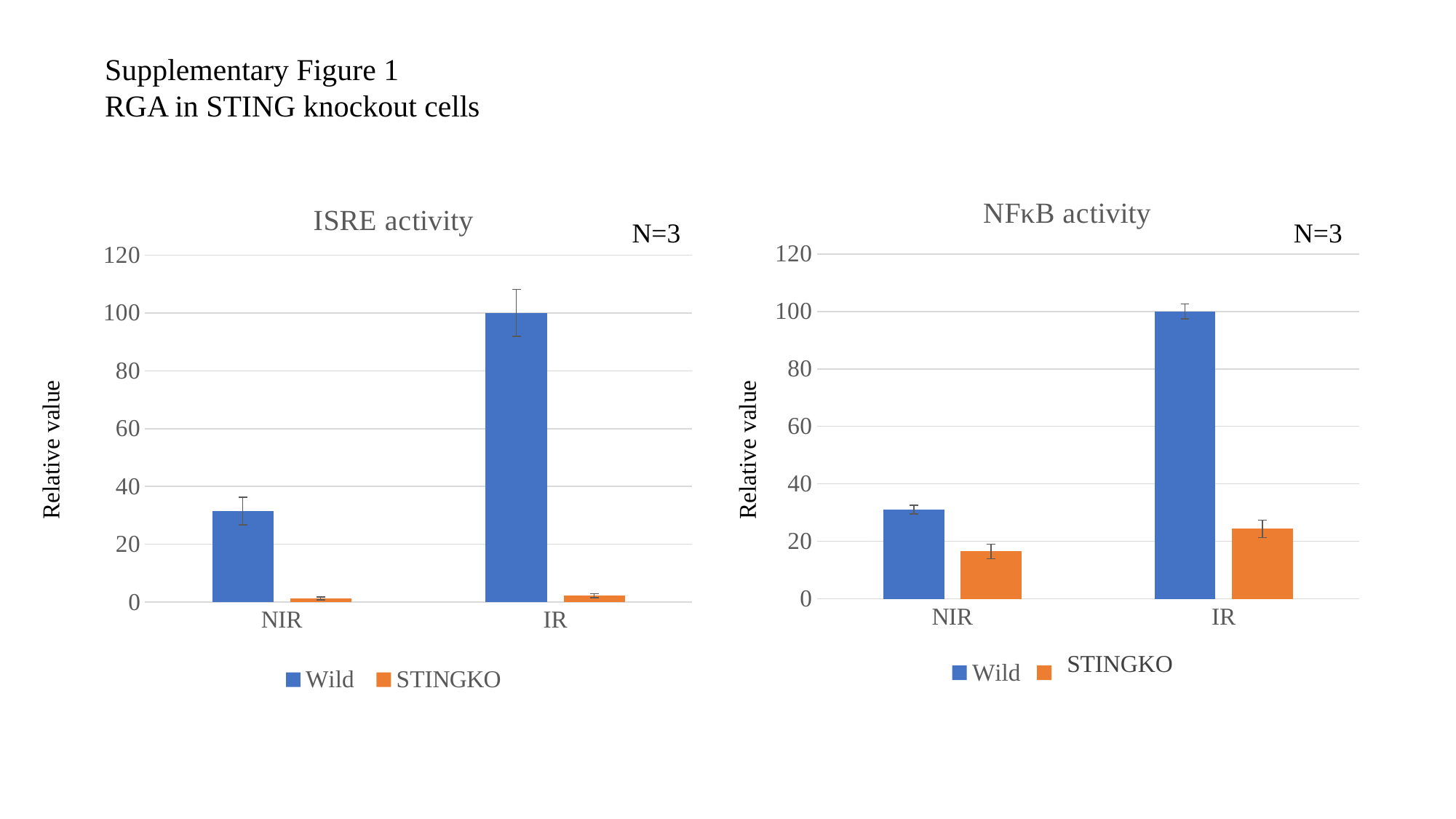

Supplementary Figure 1
RGA in STING knockout cells
### Chart: NFκB activity
| Category | Wild | STING-5KO |
|---|---|---|
| NIR | 31.048980227402257 | 16.480087562417705 |
| IR | 100.0 | 24.332591048335196 |
### Chart: ISRE activity
| Category | Wild | STINGKO |
|---|---|---|
| NIR | 31.488060671243545 | 1.3578030762447881 |
| IR | 100.0 | 2.3004113312882715 |N=3
N=3
Relative value
Relative value
STINGKO

## Slide 2
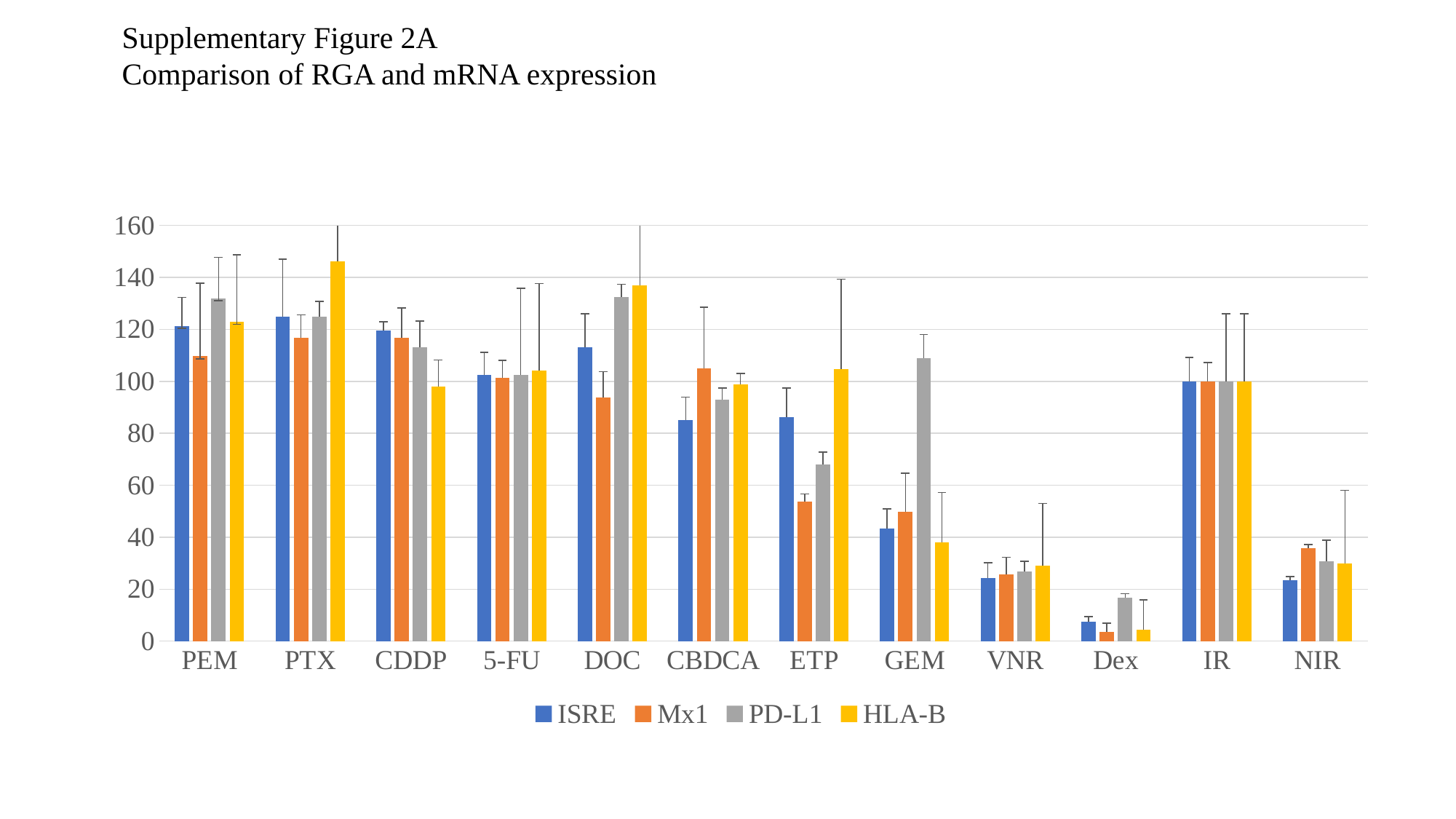

Supplementary Figure 2A
Comparison of RGA and mRNA expression
### Chart
| Category | ISRE | Mx1 | PD-L1 | HLA-B |
|---|---|---|---|---|
| PEM | 121.33333333333333 | 109.66666666666667 | 132.0 | 122.92192494087972 |
| PTX | 125.0 | 116.66666666666667 | 125.0 | 146.152321967661 |
| CDDP | 119.66666666666667 | 116.66666666666667 | 113.0 | 98.0724212879 |
| 5-FU | 102.33333333333333 | 101.33333333333333 | 102.33333333333333 | 104.05381409978652 |
| DOC | 113.0 | 93.66666666666667 | 132.33333333333334 | 136.861281261448 |
| CBDCA | 85.0 | 105.0 | 93.0 | 98.6774469399085 |
| ETP | 86.33333333333333 | 53.666666666666664 | 68.0 | 104.640925279215 |
| GEM | 43.333333333333336 | 49.666666666666664 | 109.0 | 38.082023416089 |
| VNR | 24.333333333333332 | 25.666666666666668 | 26.666666666666668 | 28.90309953550215 |
| Dex | 7.333333333333333 | 3.6666666666666665 | 16.666666666666668 | 4.338218129014376 |
| IR | 100.0 | 100.0 | 100.0 | 100.0 |
| NIR | 23.333333333333332 | 35.666666666666664 | 30.666666666666668 | 29.88888888888889 |

## Slide 3
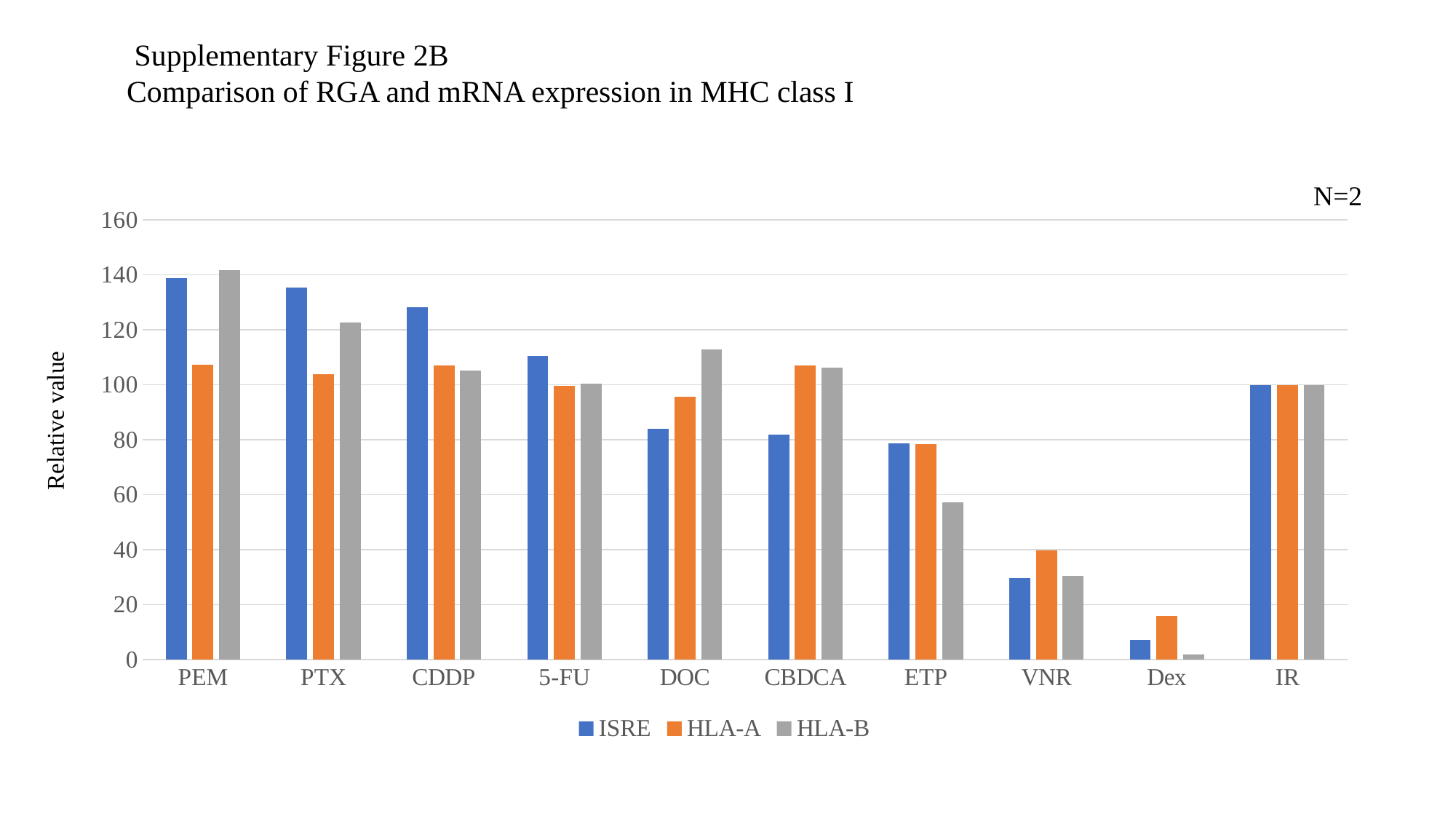

Supplementary Figure 2B
Comparison of RGA and mRNA expression in MHC class I
N=2
### Chart
| Category | ISRE | HLA-A | HLA-B |
|---|---|---|---|
| PEM | 138.8380696977088 | 107.19626097429797 | 141.73692640768837 |
| PTX | 135.46794759030587 | 103.87386090763788 | 122.57933923573829 |
| CDDP | 128.25835453122286 | 106.98794815662094 | 105.11502649041593 |
| 5-FU | 110.41646435978194 | 99.55080181497277 | 100.41363772376042 |
| DOC | 84.02491017913431 | 95.78374857021656 | 112.92133842606444 |
| CBDCA | 82.01521230765422 | 107.04854824903607 | 106.14120007744705 |
| ETP | 78.7341552077819 | 78.4134895805716 | 57.21225775790752 |
| VNR | 29.68256482682456 | 39.7362380977631 | 30.563427384577473 |
| Dex | 7.271916517523389 | 15.809806609955078 | 1.8094450213859505 |
| IR | 100.0 | 100.0 | 100.0 |Relative value

## Slide 4
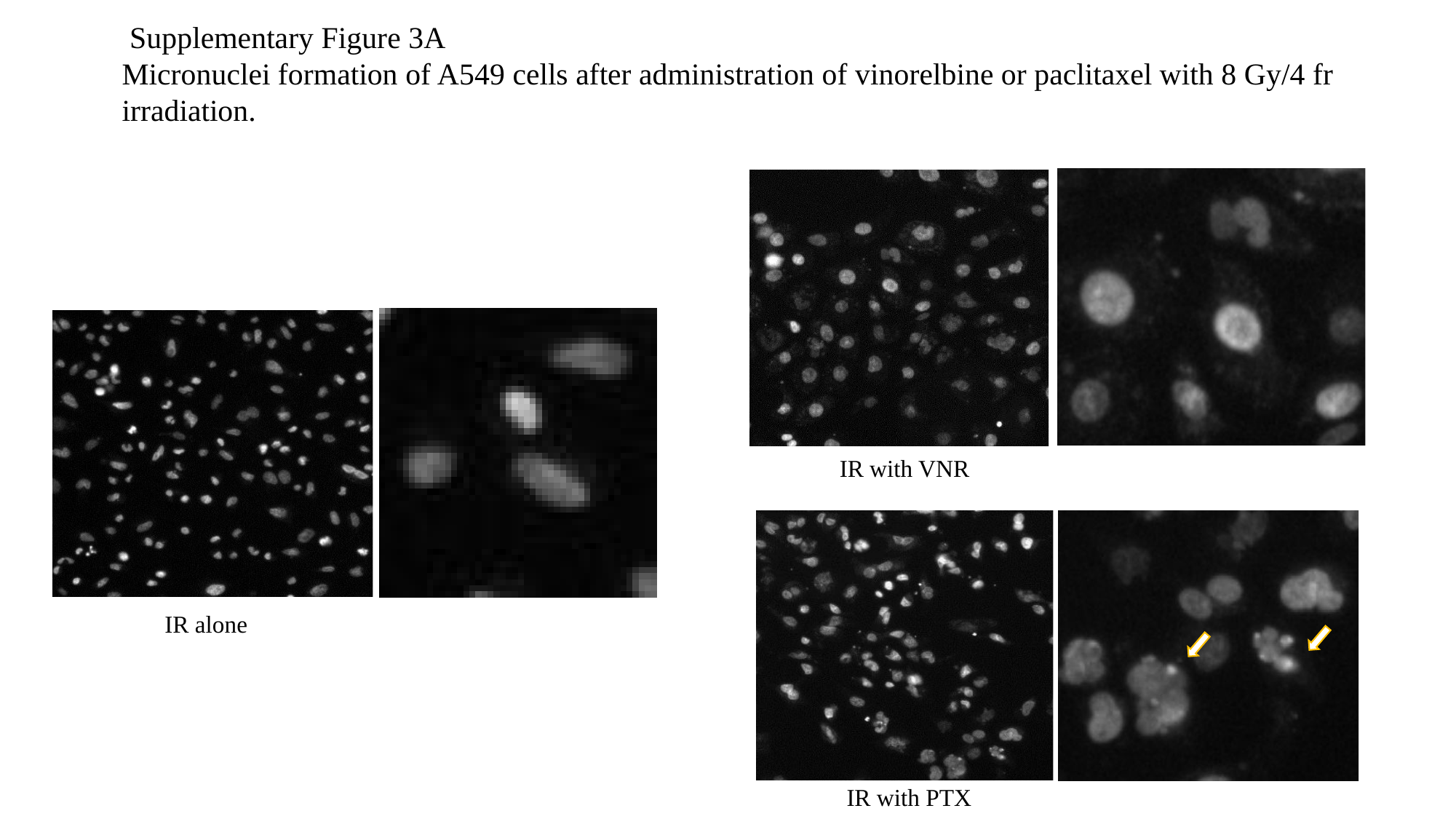

Supplementary Figure 3A
Micronuclei formation of A549 cells after administration of vinorelbine or paclitaxel with 8 Gy/4 fr irradiation.
IR with VNR
IR alone
IR with PTX

## Slide 5
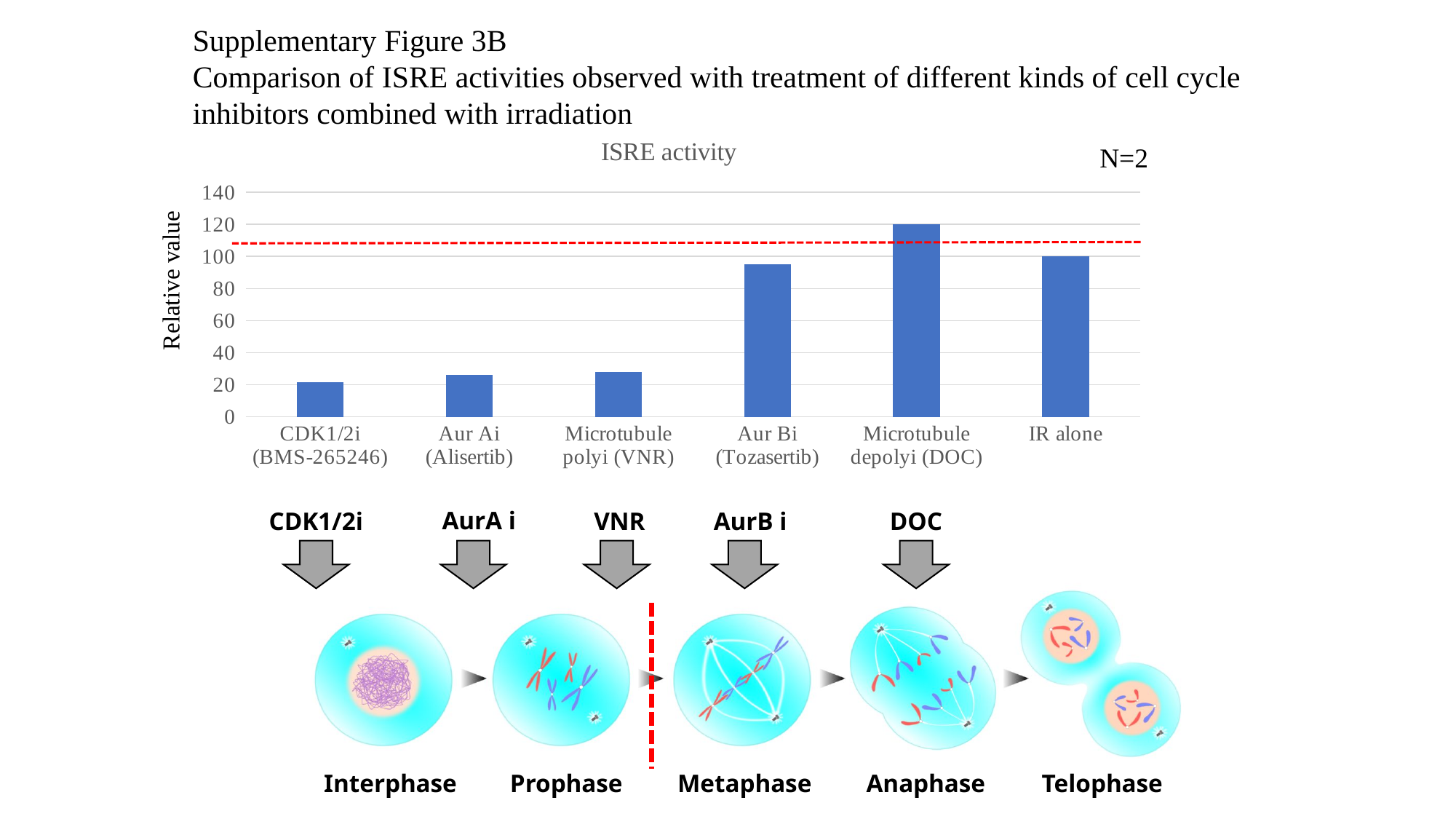

Supplementary Figure 3B
Comparison of ISRE activities observed with treatment of different kinds of cell cycle inhibitors combined with irradiation
### Chart: ISRE activity
| Category | |
|---|---|
| CDK1/2i (BMS-265246) | 21.753000080804934 |
| Aur Ai (Alisertib) | 25.976626150624156 |
| Microtubule polyi (VNR) | 27.802685199065664 |
| Aur Bi (Tozasertib) | 95.13782136905135 |
| Microtubule depolyi (DOC) | 120.06131262214468 |
| IR alone | 100.0 |N=2
Relative value
AurA i
DOC
CDK1/2i
VNR
AurB i
Interphase
Prophase
Metaphase
Anaphase
Telophase

## Slide 6
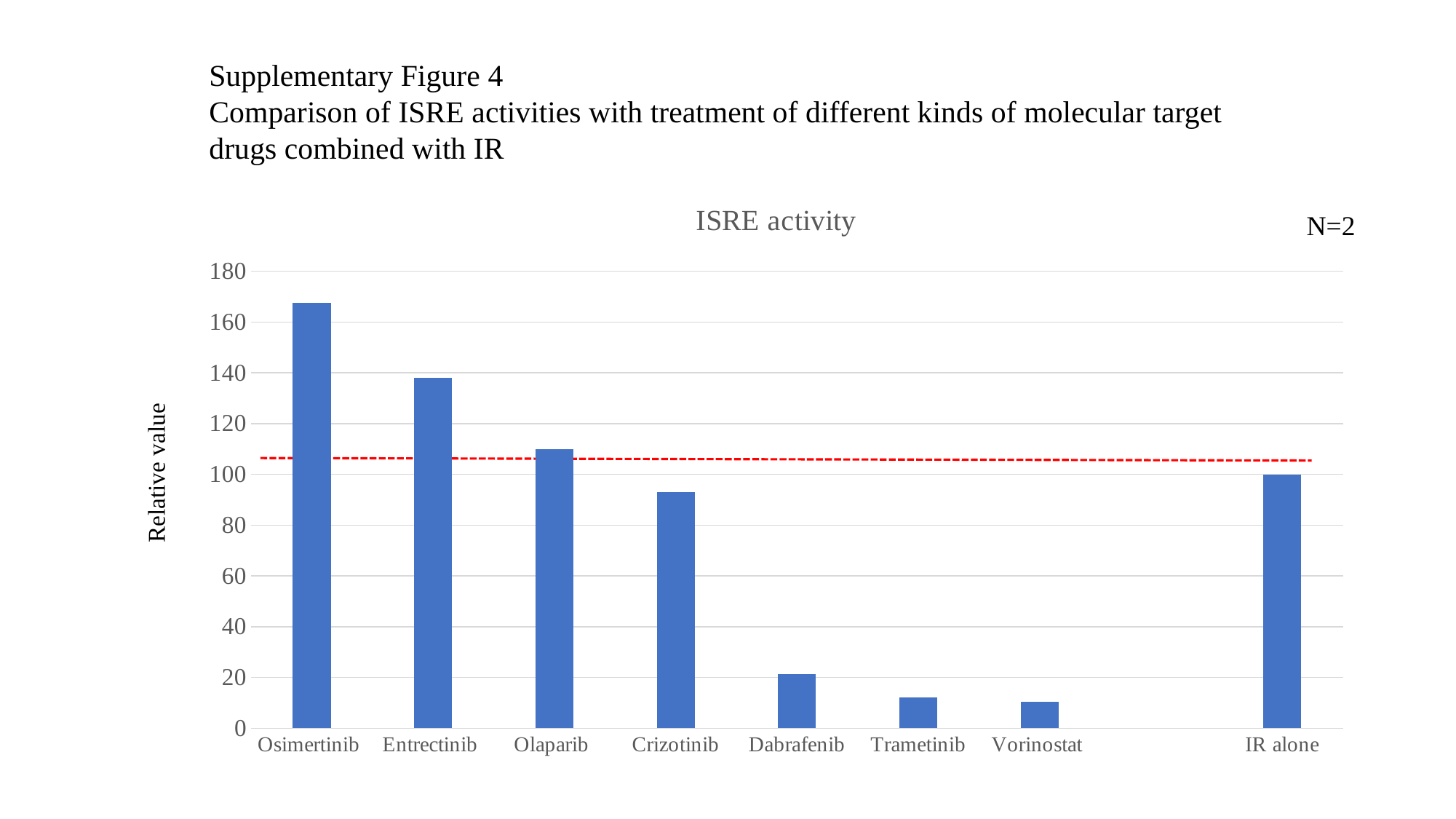

Supplementary Figure 4
Comparison of ISRE activities with treatment of different kinds of molecular target drugs combined with IR
### Chart: ISRE activity
| Category | |
|---|---|
| Osimertinib | 167.4954930137358 |
| Entrectinib | 138.0 |
| Olaparib | 110.06861784531728 |
| Crizotinib | 92.9020753541961 |
| Dabrafenib | 21.404736166924167 |
| Trametinib | 12.279697249101602 |
| Vorinostat | 10.521801589265637 |
| | None |
| IR alone | 100.0 |N=2
Relative value

## Slide 7
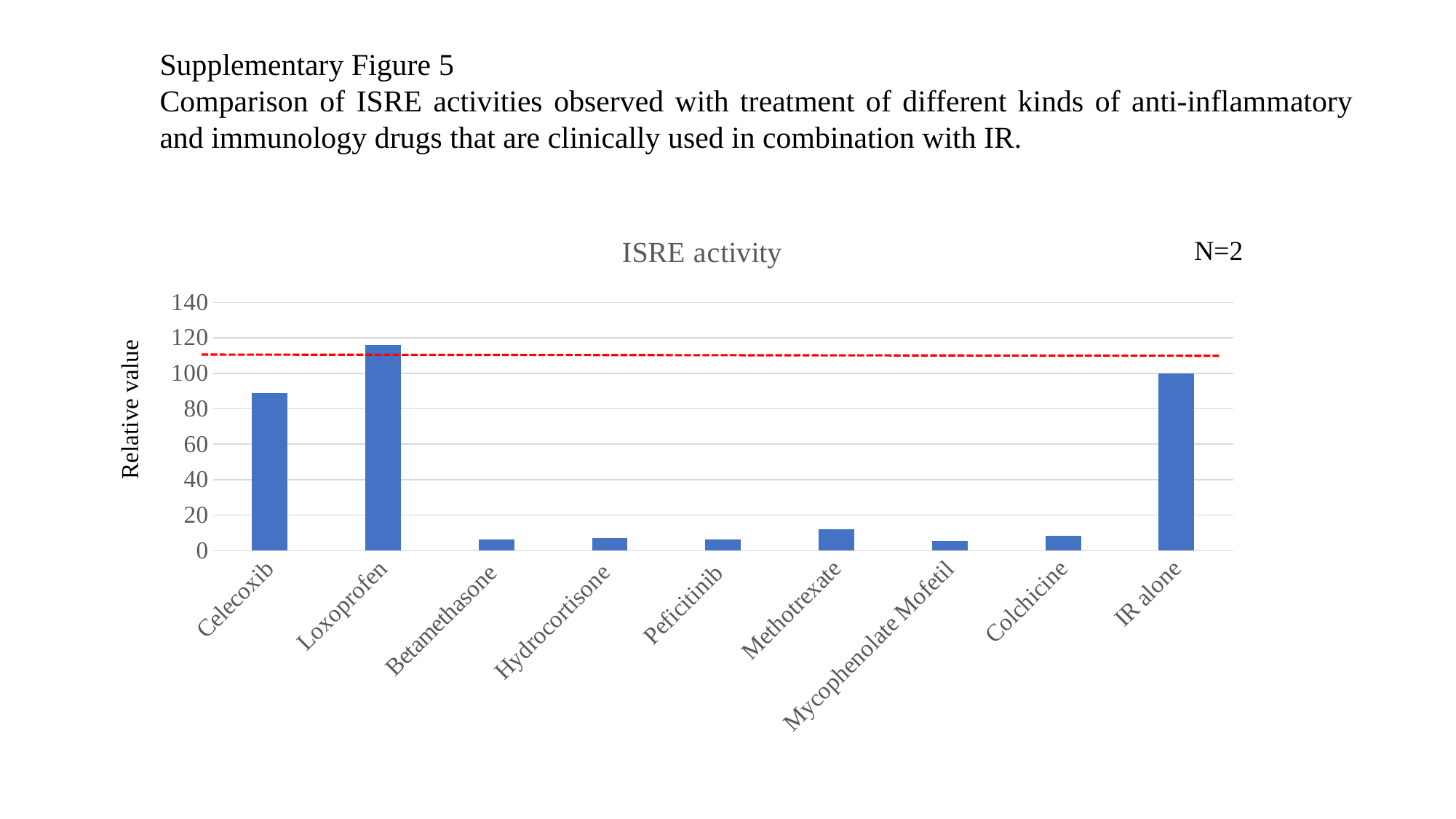

Supplementary Figure 5
Comparison of ISRE activities observed with treatment of different kinds of anti-inflammatory and immunology drugs that are clinically used in combination with IR.
### Chart: ISRE activity
| Category | ISRE activity |
|---|---|
| Celecoxib | 88.73061442345895 |
| Loxoprofen | 116.01520406752606 |
| Betamethasone | 6.415084938571317 |
| Hydrocortisone | 7.099731743589123 |
| Peficitinib | 6.291705115449743 |
| Methotrexate | 12.10376343366611 |
| Mycophenolate Mofetil | 5.365830393592275 |
| Colchicine | 8.153085159337394 |
| IR alone | 100.0 |N=2
Relative value
