# Supplementary material for: Comprehensive screening for drugs that modify radiation-induced immune responses
Source: Br J Cancer. 2022 Feb 19;126(12):1815–23. doi: 10.1038/s41416-021-01688-0 (PMC9174493; doi:10.1038/s41416-021-01688-0)
Supplement: Supplementary file 7 — Supplementary Table 4 [file 41416_2021_1688_MOESM7_ESM.docx]

| Name | Dilution buffer | Dilution ratio | ID |
| --- | --- | --- | --- |
| Anti-PD-L1 antibody [EPR19759] | 5% NFDM/TBS-T | 1:1000 | ab213524, Abcam |
| GAPDH (14C10) Rabbit mAb |  | 1:2000 | #2118, CST |
| Stat1 (D1K9Y) Rabbit mAb |  | 1:1000 | #14994, CST |
| STING (D2P2F) Rabbit mAb |  | 1:1000 | #13647, CST |
| Anti-rabbit IgG, HRP-linked Antibody |  | 1:2000 | #7074, CST |
| pStat1 (Tyr701) (D4A7) Rabbit mAb |  | 1:1000 | #7649, CST |

Supplemental Table 4
