# Supplementary material for: Comprehensive screening for drugs that modify radiation-induced immune responses
Source: Br J Cancer. 2022 Feb 19;126(12):1815–23. doi: 10.1038/s41416-021-01688-0 (PMC9174493; doi:10.1038/s41416-021-01688-0)
Supplement: Supplementary file 1 — Author’s consent English [file 41416_2021_1688_MOESM1_ESM.docx]

This is the mail to confirm the addition of authors to all authors.

差出人: 影山　俊一郎 <skageyam@east.ncc.go.jp>

日付: 月曜日, 2021年11月29日 9:02

宛先: 奥村真之 <moku1104@med.nagoya-u.ac.jp>, 杜　君顔 <jdu@east.ncc.go.jp>, 山下　理宇 <riuyamas@east.ncc.go.jp>, 箱崎　優美 <yhakozak@east.ncc.go.jp>, 茂木　厚 <amotegi@east.ncc.go.jp>, 北條　秀博 <hhojo@east.ncc.go.jp>, 中村　匡希 <masanaka@east.ncc.go.jp>, 平野　靖弘 <yahirano@east.ncc.go.jp>, 大熊　裕介 <yokuma@ncc.go.jp>, Hitomi S. OKUMA（大熊　ひとみ） <hsumiyos@ncc.go.jp>, Katsuya Tsuchihara <ktsuchih@east.ncc.go.jp>, 秋元　哲夫 <takimoto@east.ncc.go.jp>

件名: BJC掲載前のお願いAs I mentioned the other day "Comprehensive screening for drugs that modify radiation-induced immune responses" has been accepted by the British Journal of Cancer.

Dr. Hitomi Okuma and Yumi Hakozaki cooperated with Revise Response. BJC has informed us that the addition of authors after the first submit requires the consent of all authors.

Confirmation by email is OK. Please reply to this e-mail and comment for your consent to add the author of Dr. Hitomi Okuma and Yumi Hakozaki.

Best regard.

Kageyama

共著者の先生方

先日お伝えさせて頂きましたが、”Comprehensive screening for drugs that modify radiation-induced immune responses”がBritsh Journal of Cancerにアクセプトされました。

がんセンター中央病院大熊ひとみ先生と箱崎さんにリバイスレスポンスの協力を頂きました。初回submit以降の著者追加は著者全員の同意が必要とBJCから連絡がありました（大熊先生は初回から入れていたと思いますが・）。

メールでの確認でOKで、メールと取りまとめたファイルを作成し、BJCに送ります。このメールに返信で大熊ひとみ先生, 箱崎さんの著者追加の同意をお願いします。

The response from co-authors are follows.

奥村真之 <moku1104@med.nagoya-u.ac.jp>

2021/11/29 (月) 11:16

影山先生

取りまとめありがとうございます。

同意いたします。

I agree with the addition of the author.

奥村真之

Masayuki Okumura

茂木　厚

2021/11/29 (月) 10:11

影山先生

お疲れ様です。

同意いたします。

よろしくお願いいたします。

I agree with the addition of the author.

茂木

Atsushi Motegi

北條　秀博

2021/11/29 (月) 10:21

影山先生

同意いたします。

I agree with the addition of the author.

北條秀博

Hidehiro Hojo MD, PhD

中村　匡希

2021/11/29 (月) 9:26

Dr. Kageyama

I agree with the addition of the author.

Regards,

Masaki Nakamura

Radiation Oncology

National Cancer Center Hospital East

平野　靖弘

2021/12/02 (木) 5:33

影山先生

ご無沙汰しております。

メールの確認が遅れまして申し訳ございません。

著者追加に同意いたします。

よろしくお願いいたします。

I agree with the addition of the author.

平野靖弘

Yasuhiro Hirano

箱崎　優美

2021/12/02 (木) 10:36

影山先生

著者の追加に同意いたします。

I agree with the addition of the author.

箱崎優美

Yumi Hakozaki

大熊　裕介

2021/11/29 (月) 23:22

Dear Dr. Kageyama,

I agree with the addition of authors.

Best wishes,

Yusuke Okuma

Hitomi S. OKUMA（大熊　ひとみ）

2021/12/02 (木) 10:36

景山先生

大変失礼いたしました。

賛同いたします。

大熊ひとみ

I agree to the addition of authors Dr. Hitomi Okuma and Yumi Hakozaki.

Best regards,

Hitomi Okuma

National Cancer Center Hospital, Tokyo

Katsuya Tsuchihara

2021/11/29 (月) 9:13

Dear Dr. Kageyama,

Congratulations on the acceptance of the manuscript. I agree with the addition of the author.

Best regards,

Katsuya Tsuchihara

---

Katsuya Tsuchihara, MD, PhD

Chief, Division of Translational Informatics,

Exploratory Oncology Research and Clinical Trial Center,

National Cancer Center Japan

6-5-1 Kashiwanoha, Kashiwa, Chiba 277-8577, Japan

Phone: +81-4-7133-1111

Fax: +81-4-7134-8786

E-mail: [ktsuchih@east.ncc.go.jp](mailto:ktsuchih@east.ncc.go.jp)

秋元　哲夫

2021/11/29 (月) 9:21

影山先生

著者の追加に同意します。

秋元哲夫

I agree with the addition of the author.

Tetsuo Akimoto
